# Supplementary material for: Content-rich biological network constructed by mining PubMed abstracts
Source: BMC Bioinformatics. 2004 Oct 8;5:147. doi: 10.1186/1471-2105-5-147 (PMC528731; doi:10.1186/1471-2105-5-147)
Supplement: Additional File 2 — The original results of the above study (non-essential files are deleted to keep the file size under the limit set by BMC bioinformatics). [file 1471-2105-5-147-S2.bz2 › chilibotAdditionalFile2/dip20/354ID1495999E4996/html/IL5_IL5RA.html]

 


 **IL5** and **IL5RA** 
  
Found 67 abstracts in PubMed, retrieved 20.  
 

 What does Google say? 
 PDF only 
| .edu only 

---

**Interactive relationship** (e.g. stimulation, inhibition, etc)

**Neutral relationship**- Using this surface, we quantitated for the first time direct binding of s beta c to IL5R  [ **IL5RA** ]  alpha complexed to either wild type or single chain  **IL5** .  Ref: 12202242 Anal Biochem, 2002
- Biosensor analysis of dynamics of interleukin 5  [ **IL5** ]  receptor subunit beta c interaction with  **IL5**  IL5R  [ **IL5RA** ]  alpha complexes.  Ref: 12202242 Anal Biochem, 2002
- We therefore suggest that an IL 5  [ **IL5** ]  driven inflammation generates an eosinophil tissue phenotype that is characterized by a low IL 5R alpha  [ **IL5RA** ]  expression.  Ref: 12519389 Clin Exp Immunol, 2003
- The IL 5R alpha  [ **IL5RA** ]  was down regulated, in a dose dependent manner, by recombinant IL 5  [ **IL5** ]  and GM CSF, with IL 5  [ **IL5** ]  being most potent.  Ref: 12519389 Clin Exp Immunol, 2003
- Experiments with neutralizing antibodies against IL 5  [ **IL5** ]  and GM CSF, revealed that IL 5  [ **IL5** ]  was the principal cytokine responsible for the down regulation of the IL 5R alpha  [ **IL5RA** ] .  Ref: 12519389 Clin Exp Immunol, 2003
- In conclusion our data indicate that IL 5  [ **IL5** ] , as opposed to its proposed action on eosinophil progenitors, down regulates the IL 5R alpha  [ **IL5RA** ]  chain on mature eosinophils.  Ref: 12519389 Clin Exp Immunol, 2003
- These aspects of IL 5  [ **IL5** ]  action on IL 5R alpha  [ **IL5RA** ]  expression could gain new insights into the mechanisms of specific immuno modulatory therapies, such as anti IL 5  [ **IL5** ] .  Ref: 12519389 Clin Exp Immunol, 2003

**Non-interactive relationship** (e.g. studied together, co-existance, homology, etc.)

- Incubation of eosinophils with IL 3, IL 5  [ **IL5** ] , or GM CSF led to reduced expression of IL 5R alpha  [ **IL5RA** ] , which was sustained for up to 5 days.  Ref: 12759409 J Immunol, 2003
- RFX and 3 regulate expression of other medically important gene products for example, interleukin 5  [ **IL5** ]  receptor alpha chain, IL 5R alpha  [ **IL5RA** ] .  Ref: 10706293 Nature, 2000
- As part of our effort to define molecular recognition networks that lead to signalling, we investigated the effect of JAB on both JAK2 phosphorylation and JAK2 interaction state that ensue upon IL 5  [ **IL5** ]  stimulation in recombinant 293T cells cotransfected 293T cells with IL 5R alpha  [ **IL5RA** ] , beta c and hJAK2 either with or without JAB.  Ref: 10975987 Cytokine, 2000
- The results obtained with IL 5  [ **IL5** ]  knockout mice and with IL 5R alpha  [ **IL5RA** ]  knockout mice can be used to guide and inform asthma and allergy research in humans.  Ref: 11191098 Allergy Asthma Proc< MedlineTA>Allergy Asthma Proc, 2000
- The addition of IL 9 to CD34 cells cultured in IL 3 and IL 5  [ **IL5** ]  enhanced eosinophil development, and IL 9 alone induced the expression of IL 5R alpha  [ **IL5RA** ] .  Ref: 10979962 Blood, 2000
- RT PCR was used to evaluate IL 5  [ **IL5** ]  receptor alpha IL 5R alpha  [ **IL5RA** ]  expression in various tissues and to assess bronchus and saphenous vein eosinophils through use of CCR3 expression.  Ref: 11897983 J Allergy Clin Immunol, 2002
- We studied the expression of the IL 5  [ **IL5** ]  receptor alpha subunit IL 5R alpha  [ **IL5RA** ]  on bone marrow derived cluster of differentiation molecule 34 positive CD34 progenitor cells in asthmatics to prove the ability of progenitor cells to respond to IL 5  [ **IL5** ]  more readily.  Ref: 10584413 Changgeng Yi Xue Za Zhi, 1999
- The aim was to study in vitro regulation of the IL 5  [ **IL5** ]  receptor alpha IL 5R alpha  [ **IL5RA** ]  on purified peripheral blood eosinophils from healthy subjects.  Ref: 12519389 Clin Exp Immunol, 2003
- OBJECTIVE To investigate the iufluence of dexamethasone DM on expression of interleukin 5  [ **IL5** ]  receptor alpha IL 5R alpha  [ **IL5RA** ] , interleukin 3 receptor alpha IL 3R alpha, granulocyte macrophage colony stimulating factor receptor alpha GM CSFR alpha and common beta receptor beta cR in bronchalveolar lavage fluid BALF eosinophils in guinea pig asthma model, and to study the mechanism of promotion effect of steroid on eosinophil apoptosis.  Ref: 11758170 Zhonghua Jie He He Hu Xi Za Zhi, 2001
- What is the primary function of eosinophils ? Regulatory or anti inflammatory or pro inflammatory ? Results with IL 5  [ **IL5** ]  knockout mice and with IL 5R alpha  [ **IL5RA** ]  knockout mice clearly show that 1 eosinophils play key roles as cellular mediators in asthma and allergy,.  Ref: 11191098 Allergy Asthma Proc< MedlineTA>Allergy Asthma Proc, 2001
